# Comparison of Semiparametric, Parametric, and Nonparametric ROC Analysis for Continuous Diagnostic Tests Using a Simulation Study and Acute Coronary Syndrome Data

**DOI:** 10.1155/2012/698320

**Published:** 2012-06-28

**Authors:** Ertugrul Colak, Fezan Mutlu, Cengiz Bal, Setenay Oner, Kazim Ozdamar, Bulent Gok, Yuksel Cavusoglu

**Affiliations:** ^1^Department of Biostatistics and Medical Informatics, School of Medicine, Eskisehir Osmangazi University, 26480 Eskisehir, Turkey; ^2^Department of Cardiology, Private Bursa Anadolu Hospital, Izmir Yolu 105, 16120 Nilufer/Bursa, Turkey; ^3^Department of Cardiology, School of Medicine, Eskisehir Osmangazi University, 26480 Eskisehir, Turkey

## Abstract

We aimed to compare the performance of three different individual ROC methods (one from each of the broad categories of parametric, nonparametric and semiparametric analysis) for assessing continuous diagnostic tests: the binormal method as a parametric method, an empirical approach as a nonparametric method, and a semiparametric method using generalized linear models (GLM). We performed a simulation study with various sample sizes under normal, skewed, and monotone distributions. In the simulations, we used estimates of the ROC curve parameters *a* and *b*, estimates of the area under the curve (*AUC*), the standard errors and root mean square errors (RMSEs) of these estimates, and the 95% *AUC* confidence intervals for comparison. The three methodologies were also applied to an acute coronary syndrome dataset in which serum myoglobin levels were used as a biomarker for detecting acute coronary syndrome. The simulation and application studies suggest that the semiparametric ROC analysis using GLM is a reliable method when the distributions of the diagnostic test results are skewed and that it provides a smooth ROC curve for obtaining a unique cutoff value. A sample size of 50 is sufficient for applying the semiparametric ROC method.

## 1. Introduction

Receiver operating characteristic (ROC) curve analysis is used in many scientific fields to determine the accuracy of a diagnostic test, for example, in signal detection theory and medicine [1–7]. An ROC curve is a plot of the false positive rates against the true positive rates for various cutoff values of the diagnostic test result. The most commonly used value to summarize the accuracy is the *area under the ROC curve* (*AUC*). The *AUC* can take values between 0 and 1, and greater *AUC* values denote better accuracy [6]. 

The result of a diagnostic test may be binary, ordinal, or continuous. Most medical diagnostic tests, such as biomarkers for myocardial injury or cancer, have continuous test results [5, 8]. The most common ROC analyses are nonparametric. The nonparametric ROC methods do not require any assumptions about the diagnostic test result distributions and do not provide a smooth ROC curve. However, parametric methods assume that some function of the diagnostic test measurements are normally distributed in both the diseased and nondiseased populations but with different means. It is possible to obtain a smooth ROC curve using the parametric ROC methods. For comparison, we chose the empirical ROC method [9, 10] an example from the nonparametric category and the binormal ROC method, which has been popularized by Metz and other researchers [11–13], as an example from the parametric category. The empirical and binormal ROC methods are included in most major statistical packages and are the most popular methods within the nonparametric and parametric categories, respectively. The third alternative to these two traditional ROC categories is semiparametric ROC analysis. Semiparametric ROC methods do not make any distributional assumptions about the results of the diagnostic test and also yield a smooth ROC curve. The main difference between the semiparametric and nonparametric methods is that semiparametric methods can estimate ROC curve parameters without making any assumptions about the distribution of the diagnostic test results. Many semiparametric ROC approaches have been developed. For example, Gang et al. [14] developed a semiparametric ROC method using a nonparametric approach for the test result distribution of the nondiseased group and a parametric methodology for the test results of the diseased group. Cai and Moskowitz [15] proposed using two methods, profile likelihood and pseudo-maximum likelihood, to estimate the ROC curve parameters. In addition to these semiparametric approaches, Wan and Zhang [16] used a kernel distribution function estimator. In their study, Pepe's [17] application of the generalized linear model (GLM) to ROC curves was used as a semiparametric ROC method because of its highly efficient estimators. In this approach, inferences are made using binary regression techniques applied to indicator variables constructed from paired test results (one component from a diseased subject and the other from a nondiseased subject). ROC curve parameter estimates can be easily obtained using the GLM binary regression framework, and the effects of covariates can be evaluated. Thus, we chose the semiparametric method for our comparison. 

In this study, our specific objectives were to compare the performance of three different individual ROC methods for assessing a continuous diagnostic and to determine which method is efficient under which conditions. To achieve these goals, we generated simulated random datasets of various sample sizes from normal, skewed, and monotone distributions using the SAS/IML and SAS GENMOD procedure in SAS 9.1. The three methods were also applied to an actual acute coronary syndrome dataset. For comparison, we used the estimates of the ROC curve parameters *a* and *b*, the *AUC* estimates, the standard errors of these estimates, the 95% *AUC* confidence intervals, and the root mean square errors (RMSE) of *AUC* estimates. 

## 2. Materials and Methods

Let *Y* denote a random variable representing a continuous diagnostic test result. The diagnosis according to any cutoff value *c* is positive if *Y* ≥ *c* and negative if *Y* < *c*. Let *D*_0_ and *D*_1_ denote the nondiseased and diseased populations, respectively. The true and false positive rates at the cutoff value *c*, *TP*(*c*), and *FP*(*c*) are

(1) TP(c)=P(Y≥c ∣ D1),FP(c)=P(Y≥c ∣ D0).

The ROC curve is denoted by

(2) ROC(t)=1−F1(F0−1(1−t)),

where *TP*(*c*) = *F*_1_(*c*), *FP*(*c*) = *F*_0_(*c*), and *t* is the all possible *FP* rates according to the varying *c* values in (−*∞*, *∞*) [5]. 

### 2.1. Parametric ROC

In this study, the binormal method was used for the parametric ROC analysis. The main assumption of this method is that the results of the continuous diagnostic test in the diseased (*Y*_1_) and nondiseased (*Y*_0_) populations are normally distributed with different means:

(3) Y1~N(μ1,σ12),  Y0~N(μ0,σ02).

The ROC curve is modeled by the following function:

(4) ROC(t)=Φ(a+bΦ−1(t)),

where Φ is the cumulative normal distribution function, and

(5) a=μ1−μ0σ1,  b=σ0σ1,

see [11–13]. 

The *AUC* equals the probability that a randomly selected diseased subject has diagnostic higher than a randomly selected nondiseased subject:

(6) AUC=Φ(a1+b2),

where *ϕ* is the normal probability density function [5, 12, 13]. Thus, the estimates of *a*, *b*, and *AUC *(denoted by $\hat{a}$ , $\hat{b}$ , and $A\hat{U}C$ , resp.) are computed using ${\hat{\mu }}_{1}$ , ${\hat{\mu }}_{0}$ , ${\hat{\sigma }}_{1}$ , and ${\hat{\sigma }}_{0}$ . The respective variances of $\hat{a}$ and $\hat{b}$ are

(7) V(a^)=n1(a^2+2)+2n0b^22n0n1,V(b^)=(n1+n0)b^22n0n1,

where *n*_1_ and *n*_0_ are the numbers of diseased and nondiseased study subjects, respectively. The variance of $A\hat{U}C$ can be derived using the delta method [6].

### 2.2. Nonparametric ROC

In our study, the empirical method was used for the nonparametric ROC analysis. This method is popular because it does not make any distributional assumptions about the diagnostic test measurements. In this approach, the possible diagnostic test results for each cutoff value *c* are considered, and the corresponding true and false positive rates are calculated by

(8) TP(c)=s1(c)n1,FP(c)=s0(c)n0,

where *s*_1_(*c*) is the number of subjects with test results greater than or equal to *c*(*Y* ≥ *c*) among the diseased subjects and *s*_0_(*c*) is the number of subjects with test results greater than or equal *c*(*Y* ≥ *c*) among the nondiseased subjects. The ROC curve is subsequently created by connecting these points with a straight line [9, 10]. The *AUC* of the nonparametric ROC curve is obtained using trapezoidal rule and is estimated by

(9) AU^C=1n1n0∑i=1n1∑j=1n0ψ(Yi1,Yj0),

where

(10) ψ(Yi1,Yj0)={1if  Yi1>Yj012if  Yi1=Yj00if  Yi1<Yj0

and *Y*_*i*1_ and *Y*_*j*0_ are the diagnostic test results for the diseased and nondiseased individuals, respectively. The variance of the estimated *AUC* is computed using Mann-Whitney Statistic [10]:

(11) V(AU^C)=AU^C(1−AU^C)+(n1−1)(Q1−AU^C2)n1n0 +(n0−1)(Q2−AU^C2)n1n0.       

*Q*_1_ and *Q*_2_ are defined as

(12) Q1=1n0n12∑Yn0=y×[(n1>y)2+n1>y×n1=y+(n1=y)23],Q2=1n02n1∑Yn1=y×[(n0<y)2+n0<y×n0=y+(n0=y)23],

where *n*_0_^=*y*^ is the number of true negative subjects with test results equal to *y*, *n*_1_^=*y*^ is the number of true positive subjects with test results equal to *y*, *n*_0_^<*y*^ is the number of true negative subjects with test results less than *y*, and *n*_1_^>*y*^ is the number of true positive subjects with test results greater than *y* [10]. 

### 2.3. Semiparametric ROC

The semiparametric methods of ROC curve interpretation were represented by Pepe's [17] generalized linear model (GLM). Like the nonparametric method, this approach does not need to make any distributional assumptions about the diagnostic test results; similar to the parametric method, it estimates the parameters *a* and *b* and the corresponding *AUC*. Therefore, this method can be described as a semiparametric ROC analysis. To implement the semiparametric ROC approach using the GLM, a binary indicator variable is defined by

(13) Uij=I⌊Yi1≥Yj0⌋, i=1,…,n1,  j=1,…,n0

for all *n*_1_ × *n*_0_ possible pairs of diagnostic test results. Next, the false-positives rates *t*_*j*_ are calculated for all of the possible pairs using the test results of the nondiseased subjects. That is, for any pair (*Y*_*i*1_, *Y*_*j*0_), *t*_*j*_ is obtained by

(14) tj=FP(Yj0)tj∈T={1n0,…,n0n0}.

The ROC curve is constructed parametrically as

(15) g(ROCβ(t))=∑k=1Kβkhk(t),

where *g* is the specified link function, *h*_1_,…, *h*_*K*_ are basis functions, and *β*_1_,…, *β*_*K*_ are unknown parameters.

Applying GLM procedures, a linear model can be derived by using the expectation of the binary variable *U*_*ij*_ and the function *t*_*j*_. This model is defined as

(16) g(E(Uij))=∑k=1Kβkhk(tj).

If the probit link function Φ^−1^ is used, and *h*_1_(*t*_*j*_) = 1 and *h*_2_(*t*_*j*_) = Φ^−1^(*t*_*j*_), the linear model is denoted by

(17) E(Uij)=Φ(β1+β2Φ−1(tj)),

see [17]. We used probit link fuction as above because our aim is to construct the ROC model parametrically as parametric method, but then we estimate the parameters without making any assumption about diagnostic test results to make the comparisons. The parameter estimates ${\hat{\beta }}_{1}$ and ${\hat{\beta }}_{2}$ are calculated using the generalized linear model binary regression framework and can be used for $\hat{a}$ and $\hat{b}$ , respectively. The *AUC* for the semiparametric model is estimated using ${\hat{\beta }}_{1}$ and ${\hat{\beta }}_{2}$ :

(18) AU^C=Φ(β^11+β^22).

The variances of ${\hat{\beta }}_{1}$ , ${\hat{\beta }}_{2}$ and $A\hat{U}C$ are computed using bootstrap techniques [17, 18].

### 2.4. Simulation Algorithm

To compare the performances of the parametric, nonparametric, and semiparametric ROC methods, we generated continuous datasets from the normal, lognormal, and uniform distributions and applied the following simulation steps.

(1) The normally distributed diagnostic test results were generated from the normal distributions of both the diseased and nondiseased subjects (specifically *Y*_1_ ~ *N*(*a*/*b*, 1/*b*) and *Y*_0_ ~ *N*(0,1), where *a* = 1.400, *b* = 0.900), and the corresponding *AUC*≅0.850. Next, the three ROC methods were applied to this dataset, and the parameter estimates and *AUC*s (with their standard errors, RMSEs, and 95% confidence intervals) obtained from the methods were recorded.

(2) To represent diagnostic test results from a skewed distribution, a dataset was generated from the lognormal distribution for both the diseased and nondiseased subjects: *Y*_1_~Lognormal (*L* = *a*/*b*, *S* = 1/*b*) and *Y*_0_~Lognormal (*L* = 0, *S* = 1), where *L* and *S* are the location and scale parameters of the lognormal distribution and where *a* = 1.400 and *b* = 0.900 when the corresponding *AUC*≅0.850. As in step 1, the methods were applied to this dataset, and the parameter estimates and *AUC*s (with their standard errors, RMSEs, and 95% confidence intervals) were recorded.

(3) To represent diagnostic test results from a monotone distribution, datasets for both the diseased and nondiseased subjects were generated from the uniform distribution:

(19) Y1~Uniform(l=2(a/b)−(1/b)122,       r=2(a/b)+(1/b)122),Y0~Uniform(l=−3,r=3),

where *l* is the left location parameter and *r* is the right location parameter and where *a* = 1.400 and *b* = 0.900 when the corresponding *AUC*≅0.850. As in steps (1) and (2), the methods were applied to this data set, and the parameter estimates and *AUC* *s* (with their standard errors, RMSEs, and 95% confidence intervals) were recorded.

(4) The first three steps were independently replicated 1000 times. Thus, 1000 different parameter estimates and *AUC*s with their standard errors, RMSEs, and 95% confidence intervals were obtained for each method and each diagnostic test result distribution.

(5) The means of the 1000 different parameter estimates and *AUC*s with their standard errors, RMSEs, and 95% confidence intervals were calculated.

The three ROC methods were compared by evaluating how close the means of the parameter estimates were to the values determined for *a*, *b*, and *AUC*.

The various sample sizes were determined to be 30(*n*_1_ = 15, *n*_0_ = 15), 50(*n*_1_ = 25, *n*_0_ = 25), 100(*n*_1_ = 50, *n*_0_ = 50), and 200(*n*_1_ = 100, *n*_0_ = 100). The simulations and analyses were performed using the SAS/IML and SAS GENMOD procedures in SAS 9.1.

### 2.5. Application Data

**Figure 1:**
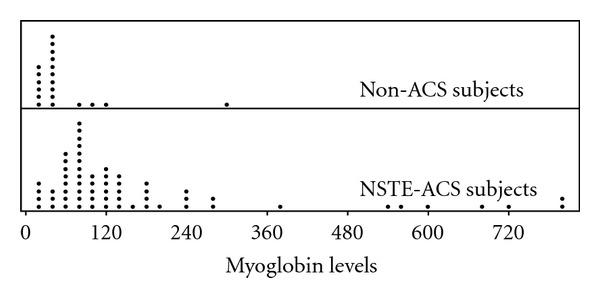
The distribution plot of the myoglobin levels.

The data set consisted of 62 patients who had been diagnosed with non-ST elevation acute coronary syndrome (NSTE-ACS) on the basis of an acute chest pain episode and electrocardiographic changes manifested by ST depressions or T wave inversions within 12 h of the symptom onset. The levels of cardiac troponin-I (cTnI) and the MB isoenzyme of creatine kinase (CK-MB) were measured at the time of emergency department arrival. A single test for myoglobin was obtained if the cTnI level was elevated. A non-ACS group consisted of 20 subjects who had atypical chest pain with normal cTnI and normal CK-MB levels. Myoglobin levels were obtained from both the NSTE-ACS and non-ACS groups.  Figure 1 shows the distribution plot of the myoglobin levels for the NSTE-ACS and non-ACS groups. The parametric, nonparametric, and semiparametric ROC analyses were applied to the data set, with the myoglobin levels serving as a biomarker for detecting ACS. Next, the results of the three ROC methods were compared. The study was approved by the Eskisehir Osmangazi University School of Medicine Ethics Committee, and the data set was collected between October 4, 2004 and September 4, 2005.

## 3. Results and Discussion

Table 1 shows the simulation results when the distributions of the continuous diagnostic test measurements were normal in both the diseased and nondiseased subjects. As the total sample size increased (especially to over 50), both the parametric and semiparametric methods provided parameter estimates with negligible bias and similar standard errors. Additionally, the three methods had similar estimates with negligible bias for the *AUC*. The standard errors, RMSE's, and 95% confidence intervals for the *AUC*'s of each method are similar with negligible differences at larger sample sizes.

Table 2 shows the diagnostic test simulation results when using a skewed distribution for the diseased and nondiseased subjects. The parametric method yielded biased parameters and *AUC* estimates at each sample size. However, the semiparametric method provided parameter and *AUC* estimates with a negligible bias when the sample size increased. The nonparametric method produced negligible *AUC* bias at each sample size. When the sample size increases, the nonparametric and semiparametric *AUC* estimates and their standard errors become similar. On the other hand, nonparametric and semiparametric methods have similar RMSE for the *AUC* estimates at each sample size. For small sample sizes, the 95% *AUC* confidence intervals from the nonparametric method have a narrower range than those of the semiparametric method.

The diagnostic test simulation results using a monotone distribution for the diseased and nondiseased subjects are shown in Table 3. These results indicate that the parametric method gave parameter estimates with negligible bias for each sample size. However, the semiparametric method provided estimates for the *a* parameter with negligible bias for smaller samples and larger bias for larger sample sizes. Standard errors of the parameter estimates were similar at larger sample size for the parametric and semiparametric method. The parametric method provided less biased *AUC* estimates at each sample size than did the semiparametric and nonparametric methods. The *AUC *estimates of each method had similar standard errors. However, the parametric method has smaller RMSE for the *AUC* estimates than the other two ROC methods. Additionally, the parametric method has narrower 95% *AUC* confidence intervals.

Table 4 indicates that the myoglobin levels are skewed and nonnormally distributed for each group. 

**Figure 2:**
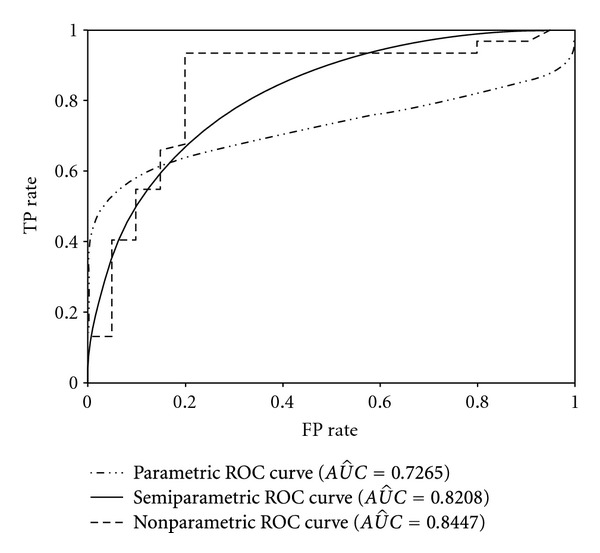
Parametric, semiparametric and nonparametric ROC curves based on the ACS data.

Table 5 shows the results of applying the three ROC methods to the ACD data set. These results were similar to the results in Table 2. The ROC curves obtained from the ACS dataset for each method are shown in Figure 2.

The semiparametric ROC method is alternative to the traditional parametric and nonparametric ROC methods. The parametric method has the restriction about the distribution of a diagnostic test which must be normal or a transformation of the test must be normal. On the other hand, the nonparametric method has a disadvantage that it does not yield smooth curve, especially in small samples. However, the semiparametric method has no assumption about the distribution of a diagnostic test and also yield smooth curve. In this case, the performances of the semiparametic method according to the other two methods were investigated and compared in this study. This paper argues that semiparametric ROC analysis using GLM is a reliable method that can be used instead of parametric and nonparametric ROC methods for continuous diagnostic test results with skewed distributions and sample sizes greater than 50. Additionally, the semiparametric ROC analysis yields a smooth ROC curve, which is important when determining a unique optimal cutoff value. 

As shown in the results of the simulation and application studies, the parametric method yielded unreliable, biased, and inconsistent parameter and *AUC* estimates when the distribution of the diagnostic test results was skewed from normality. We can conclude that applying the parametric ROC method has important restrictions. However, the simulation studies showed that the parametric ROC method can be used for diagnostic test results with monotone distributions because the results were similar to those obtained from the normally distributed diagnostic test results. In addition, the nonparametric ROC analysis yielded reliable, unbiased, and consistent estimates for the *AUC*. However, the ROC curve obtained from the nonparametric ROC analysis was not smooth. Consequently, the clinical sensitivities and specificities of a diagnostic test would vary significantly with small variations in the cutoff values. Additionally, determining a unique optimal cutoff value for a diagnostic test using a jagged ROC curve is notably difficult in real clinical applications [7, 18]. A smooth nonparametric estimation of the ROC curve can be achieved by applying kernel smoothing, and it was demonstrated that the smooth nonparametric ROC curve is superior to the jagged ROC curve in terms of deficiency [19–21]. Although this estimator is smooth and robust, the approach is not as efficient as other nonparametric ROC methods [16]. 

Our study chose to use the semiparametric model using GLM proposed by Pepe [17] and Alonzo and Pepe [18] for comparison purposes because the estimator of this method has a high statistical efficiency [15, 17, 18]. The simulation and application studies demonstrated that the semiparametric GLM method provided reliable, unbiased, and consistent estimates for the parameters and *AUC* when the sample size was over 50. Additionally, it yielded a smooth ROC curve. This result was also confirmed by the application study. 

## 4. Conclusions

We propose using the semiparametric GLM ROC method to assess the accuracy of a continuous diagnostic test if the test results have a skewed distribution. The robust estimators of this method provide a smooth ROC curve, which is important when determining the optimal cutoff value. The model also has the advantage of being easy to implement in certain statistical packages. If the results of a continuous diagnostic test have a rigorous normal or monotone distribution in both the diseased and nondiseased groups, however, the parametric method should be used. Alternatively, the semiparametric ROC method can be used for large sample sizes with normally distributed diagnostic test results. The parametric method is unreliable under other circumstances, even when the data are nearly normal. In this situation, determining the optimal cutoff value is best achieved using the semiparametric model because it has a smooth ROC curve. When applying the semiparametric ROC method, a total sample size 50 is adequate for obtaining reliable unbiased estimates and a smooth ROC curve.

This study has some limitations. The main issue was that the simulations were performed by using continuous diagnostic test results. However, comparisons can be extended including ranked data as a diagnostic test. Campbell and Ratnaparkhi [22] used Lomax distribution as a model for rating data in ROC analysis. Metz et al. [23] proposed some algorithms including truth-state runs in ranked data in ROC analysis. These models may work well for the nonnormal data. Future researches should take into account the models for comparisons.

## Figures and Tables

**Table 1:** Means of the parameter estimates and *AUC's* with their standard errors, RMSE, and 95% confidence intervals (CI) obtained from the parametric, semiparametric, and nonparametric ROC methods using various sample sizes from 1000 simulated data sets generated from the normal distribution.

		Parameters								
		*a* = 1.400, *b* = 0.900 and *AUC* = 0.850								
*n*_*1*_ * : n*_*0*_	Methods	â	SE(â)	$\hat{b}$	SE($\hat{b}$)	AÛC	BIAS for *AUC*	*SE *AÛC	RMSE for AÛC	95% CI for *AUC*
	P	1.465	0.451	0.939	0.242	0.844	0.006	0.067	0.069	0.713–0.976
15* *:* *15	S	1.578	0.507	1.170	0.302	0.822	0.028	0.072	0.075	0.681–0.963
	N	—	—	—	—	0.849	0.001	0.070	0.071	0.712–0.986

	P	1.432	0.341	0.911	0.182	0.847	0.003	0.052	0.053	0.745–0.949
25* *:* *25	S	1.446	0.356	1.007	0.202	0.834	0.016	0.052	0.058	0.731–0.937
	N	—	—	—	—	0.850	0.000	0.054	0.054	0.743–0.956

	P	1.429	0.240	0.914	0.129	0.850	0.000	0.037	0.037	0.778–0.922
50* *:* *50	S	1.430	0.245	0.966	0.137	0.843	0.007	0.037	0.039	0.771–0.915
	N	—	—	—	—	0.852	0.002	0.038	0.038	0.776–0.927

	P	1.411	0.168	0.905	0.091	0.850	0.000	0.026	0.027	0.799–0.901
100* *:* *100	S	1.409	0.170	0.932	0.093	0.846	0.004	0.026	0.027	0.795–0.897
	N	—	—	—	—	0.851	0.001	0.027	0.027	0.797–0.904

P: parametric, S: semiparametric, N: nonparametric.

**Table 2:** Means of the parameter estimates and *AUC's* with their standard errors, RMSE, and 95% confidence intervals (CIs) from the parametric, semiparametric, and nonparametric ROC methods using various sample sizes from 1000 simulated datasets generated from the lognormal distribution.

		Parameters								
		*a* = 1.400, *b* = 0.900 and *AUC* = 0.850								
*n*_*1*_ * : n*_*0*_	Methods	â	SE(â)	$\hat{b}$	SE($\hat{b}$)	AÛC	BIAS for *AUC*	*SE *AÛC	RMSE for A ÛC	95% CI for *AUC*
	P	0.729	0.303	0.236	0.061	0.754	0.096	0.088	0.113	0.581–0.928
15* *:* *15	S	1.600	0.511	1.176	0.304	0.822	0.028	0.224	0.079	0.383–1.261
	N	—	—	—	—	0.848	0.002	0.070	0.073	0.712–0.986

	P	0.693	0.230	0.216	0.043	0.746	0.104	0.067	0.116	0.610–0.882
25* *:* *25	S	1.461	0.358	1.020	0.204	0.835	0.015	0.109	0.059	0.622–1.048
	N	—	—	—	—	0.851	0.001	0.054	0.056	0.745–0.957

	P	0.646	0.159	0.196	0.028	0.734	0.116	0.050	0.127	0.635–0.832
50* *:* *50	S	1.420	0.243	0.955	0.135	0.843	0.007	0.058	0.038	0.728–0.957
	N	—	—	—	—	0.851	0.001	0.038	0.038	0.776–0.926

	P	0.601	0.111	0.183	0.018	0.721	0.129	0.036	0.133	0.650–0.792
100* *:* *100	S	1.406	0.169	0.930	0.093	0.846	0.004	0.037	0.026	0.773–0.920
	N	—	—	—	—	0.851	0.001	0.027	0.026	0.797–0.904

P: parametric, S: semiparametric, N: nonparametric.

**Table 3:** Means of the parameter estimates and *AUC'*s with their standard errors, RMSE, and 95% confidence intervals (CI) obtained from the parametric, semiparametric and nonparametric ROC methods using various sample sizes from 1000 simulated data sets generated from the uniform distribution.

		Parameters								
		*a* = 1.400, *b* = 0.900 and *AUC* = 0.850								
*n*_*1*_ * : n*_*0*_	Methods	â	SE(â)	$\hat{b}$	SE($\hat{b}$)	AÛC	BIAS for *AUC*	*SE *AÛC	RMSE for AÛC	95% CI for *AUC*
	P	1.434	0.440	0.906	0.234	0.847	0.003	0.067	0.067	0.715–0.978
15* *:* *15	S	1.402	0.474	1.130	0.292	0.810	0.040	0.068	0.084	0.677–0.943
	N	—	—	—	—	0.837	0.013	0.073	0.077	0.694–0.980

	P	1.418	0.338	0.907	0.181	0.847	0.003	0.052	0.050	0.745–0.950
25* *:* *25	S	1.373	0.353	1.060	0.212	0.820	0.030	0.053	0.062	0.716–0.923
	N	—	—	—	—	0.835	0.015	0.057	0.056	0.723–0.947

	P	1.413	0.238	0.902	0.128	0.850	0.000	0.037	0.036	0.777–0.922
50* *:* *50	S	1.357	0.242	0.992	0.140	0.829	0.021	0.038	0.045	0.755–0.902
	N	—	—	—	—	0.835	0.015	0.040	0.044	0.756–0.915

	P	1.402	0.167	0.908	0.090	0.850	0.000	0.026	0.024	0.798–0.901
100* *:* *100	S	1.350	0.169	0.967	0.097	0.832	0.018	0.027	0.033	0.780–0.885
	N	—	—	—	—	0.834	0.016	0.029	0.032	0.778–0.891

P: parametric, S: semiparametric, N: nonparametric.

**Table 4:** The descriptive statistics and normality test results for the myoglobin levels in the ACS data set.

Groups	*n*	Mean	SD	Median	Minimum	Maximum	Shapiro-Wilk test of normality
NSTE-ACS	62	178.03	194.27	104	20	800	*P* < 0.001
Non-ACS	20	54.75	64.29	33.95	11.60	304	*P* < 0.001

**Table 5:** The results of applying the parametric, semiparametric, and nonparametric ROC methods to the ACS data set.

*n*_*1*_ * : n*_*0*_	Methods	â	SE(â)	$\hat{b}$	SE($\hat{b}$)	AÛC	SE(AÛC)	95% CI for *AUC*
	Parametric	0.635	0.249	0.331	0.060	0.727	0.049	0.630–0.823
62 : 20	Semiparametric	1.310	0.331	1.018	0.185	0.821	0.030	0.761–0.880
	Nonparametric	—	—	—	—	0.845	0.044	0.759–0.930
